# Noncancer-Related Health Events and Mortality in Head and Neck Cancer Patients After Definitive Radiotherapy: A Prospective Study: Erratum

**DOI:** 10.1097/01.md.0000484756.27181.57

**Published:** 2016-06-17

In the article “Noncancer-Related Health Events and Mortality in Head and Neck Cancer Patients After Definitive Radiotherapy: A Prospective Study”,^1^ which appeared in Volume 95, Issue 19 of *Medicine*, Dr. Sang-wook Lee's name was mistakenly included in the author byline. The article has since been corrected online.
